# ESCOVE: Energy-SLA-Aware Edge–Cloud Computation Offloading in Vehicular Networks

**DOI:** 10.3390/s21155233

**Published:** 2021-08-02

**Authors:** Leila Ismail, Huned Materwala

**Affiliations:** 1 Intelligent Distributed Computing and Systems (INDUCE) Research Laboratory, Department of Computer Science and Software Engineering, College of Information Technology, United Arab Emirates University, Al Ain, Abu Dhabi 15551, United Arab Emirates; huned.m@uaeu.ac.ae; 2 National Water and Energy Center, United Arab Emirates University, Al Ain, Abu Dhabi 15551, United Arab Emirates

**Keywords:** cloud computing, computation offloading, deadline, edge computing, energy-efficiency, latency, service level agreement, quality of service, queuing theory, vehicular network

## Abstract

The vehicular network is an emerging technology in the Intelligent Smart Transportation era. The network provides mechanisms for running different applications, such as accident prevention, publishing and consuming services, and traffic flow management. In such scenarios, edge and cloud computing come into the picture to offload computation from vehicles that have limited processing capabilities. Optimizing the energy consumption of the edge and cloud servers becomes crucial. However, existing research efforts focus on either vehicle or edge energy optimization, and do not account for vehicular applications’ quality of services. In this paper, we address this void by proposing a novel offloading algorithm, ESCOVE, which optimizes the energy of the edge–cloud computing platform. The proposed algorithm respects the Service level agreement (SLA) in terms of latency, processing and total execution times. The experimental results show that ESCOVE is a promising approach in energy savings while preserving SLAs compared to the state-of-the-art approach.

## 1. Introduction

Ever-increasing vehicular traffic has led to several global issues, such as increasing incidents of road accidents, time-consuming traffic congestions, inefficient fuel utilization, and environmental impact (for example, global warming). To make travel more efficient and safe, vehicular ad hoc networks (VANETs) [1] have been introduced. Mobile vehicles, acting as network nodes, are equipped with computation and communication resources. This enables vehicles to process computational tasks aiding traffic control, efficient fuel utilization, and infotainment services. However, the computational and communication capabilities of mobile vehicles are often limited. Consequently, the performance of compute-intensive services that require real-time responses is compromised. To address this, a cloud-based vehicular network has been introduced.

Cloud computing [2] enables on-demand provisioning of computation and communication resources to the vehicles over the Internet. The vehicles’ requests tap into the scalable cloud computing resources to improve the quality-of-service (QoS), such as total execution time, processing time, throughput, and latency. However, the cloud servers located at the end of the vehicular network degrade requests’ performance due to introduced network latency. Consequently, the guarantee of QoS becomes questionable. The delay in the request-response can be life threatening in vehicular networks. For instance, a delay in the response might lead to an accident in autonomous driving. To meet the requirements of real-time and compute-intensive requests in vehicular networks, mobile edge computing (MEC) [3] has been introduced. MEC provides computing capabilities at the edge of the radio access network close to the mobile vehicles.

MEC pushes the applications and the related services from the distant cloud to the proximity of the vehicles at the edge of the network. MEC servers, deployed within the roadside units (RSUs) in a vehicular network, lead to improved QoS of the vehicles’ requests. This is because the communication latency between the MEC servers (also known as edge servers) and the vehicles is lower compared to that between the cloud servers and the vehicles. However, the edge servers are often becoming bottlenecks with the increasing and rapid services’ demands from the vehicles. This is due to the limited capabilities of the edge servers compared to the cloud ones. Therefore, it becomes necessary to offload requests to edge or cloud servers so that the performance of requests is not compromised [4].

Several works in the literature have proposed computation-offloading algorithms for vehicular networks. However, very few works focus on optimizing energy consumption. These works focus on optimizing the energy of either the IoT or the edge nodes while offloading tasks. It is important to focus on the energy efficiency of the edge and the cloud servers. This is because the energy consumption of these servers, serving computationally intensive and time-critical applications, is continuously increasing [5]. To the best of our knowledge, no work in the literature has focused on the energy consumption of the edge and cloud servers, simultaneously, while proposing an offloading algorithm.

In this paper, we propose the Energy-SLA-Aware Edge–cloud Computation Offloading in Vehicular Networks (ESCOVE) algorithm. The algorithm schedules a vehicle’s request either on the edge server, to which the request has been submitted, or to one of the cloud servers. The offloading decision is made in a way that the energy consumption for a request’s execution is minimized, while the request’s service level agreement (SLA) is respected. In this paper, we consider requests’ latencies, processing times, and deadlines (i.e., total execution time) as measures for the SLA. To respect the SLA, requests should be executed within the maximum tolerable latency, processing time, and deadline.

The rest of the paper is organized as follows. Section 2 presents an overview of related work. We describe the edge–cloud computing system model for vehicular networks in Section 3. Section 4 presents our proposed energy-efficient edge–cloud computation offloading algorithm in vehicular networks (ESCOVE). We describe the experimental environment, experiments, and analysis of our results in Section 5. Section 6 concludes the paper.

## 2. Related Work

Several works in the literature proposed computation offloading algorithms in edge–cloud vehicular networks [6,7,8,9,10,11,12,13]. However, very few works have focused on the optimization of energy consumption while considering the SLA requirements [10,11,12,13].

Ning et al. [10] proposed a computation offloading framework to partially offload computational requests to edge servers. A request is either executed on the edge server to which it has been submitted or is offloaded to other edge servers. The offloading decision is made in a way that the energy consumption of the edge servers is minimized while satisfying the requests’ deadline requirements. The total delay for processing a request is calculated as the summation of the request transmission time from vehicle to RSU, the execution time on the RSU (waiting + service), and the request transmission time among RSUs. The energy consumption is calculated as the summation of the energy consumed for request execution, transmission among RSUs, and transmission of response from RSU to the vehicle. Huang et al. [11] proposed an offloading algorithm to process requests either locally on the vehicle or to offload them to the edge server. The offloading decision is made in a way that the vehicle’s energy consumption is minimized while respecting the request’s deadline. The speed of the vehicle and the type of request is considered while computing the deadline and energy consumption. Request delay for local computing is calculated as the execution time required to process a request by the vehicle. The delay for edge computing is computed as the summation of the request transmission time between the vehicle and the edge, the processing time at the edge, and the response transmission time between the edge and the vehicle. The energy consumption for local computing is computed for processing by the vehicle and, for offloading, is computed for the transmission of the request and the response.

Huang et al. [12] proposed an offloading scheme to process requests either locally on the vehicle or to schedule them to an edge server. The offloading decision considers the energy consumption of the vehicles in terms of computation and request transmission, and the packet drop rate. Pu et al. [13] proposed an offloading algorithm to process the request either locally on the vehicle, or by a group of communicating vehicles, or by the edge server. The proposed algorithm focuses on optimizing the energy consumption of the vehicles involved in the computation process. The optimization is constrained to the request’s deadline and the incentives received by the vehicles for sharing their computational resources.

However, these works [10,11,12,13] focus on two-tiered architectures, which consist of vehicles as one tier and the MEC-enabled RSUs as the second tier. Moreover, the offloading algorithm proposed in [10] focuses only on the energy optimization of the edge servers, whereas the ones proposed in [11,12,13] focus on the energy optimization of the vehicles alone. Furthermore, no work considers more than one SLA requirement.

To the best of our knowledge, no work in the literature focuses on energy-efficient computation offloading in a three-tiered (vehicle–edge–cloud) heterogeneous vehicular network while respecting the SLA. In this paper, we propose ESCOVE to offload the vehicles’ requests either to the edge or to the cloud server. The proposed algorithm schedules requests in a way that the energy consumption of a request is minimized while satisfying the SLA requirements. In this paper, we consider request latency, processing time, and deadline as measures for SLA.

## 3. System Model

**Figure 1:**
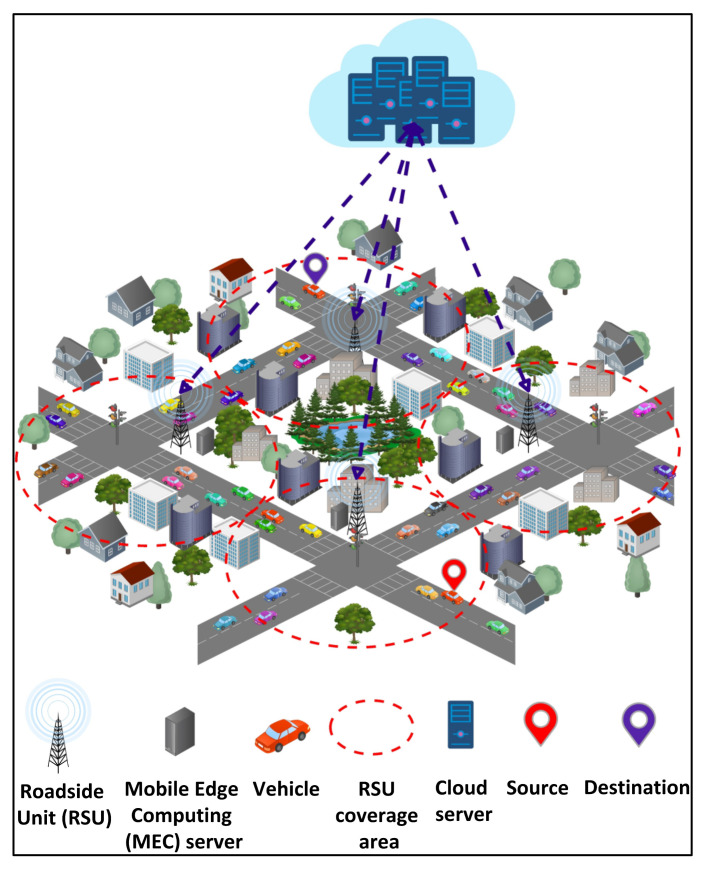
Edge–cloud system model for vehicular networks.

Figure 1 shows an edge–cloud system model for vehicular networks. It consists of several vehicles $\left(1,\;2,\;3,\;\ldots ,\;n\right)$, heterogeneous edge servers $\left(1,\;2,\;3,\;\ldots ,\;o\right)$, and heterogeneous cloud servers $\left(1,\;2,\;3,\;\ldots ,\;p\right)$. The network involves bidirectional traffic flow. The RSUs, with a limited coverage range, are placed along the road. The MEC server within the RSU processes vehicles’ requests. A vehicle can communicate with an RSU only if it is in the coverage range of that RSU. The distance between two RSUs is $D_{R}$. The processing capabilities and storage capacities of the cloud servers are higher than those of the edge servers are. However, the edge servers ensure low latency compared to the cloud servers, enabling real-time processing. This is due to the proximity between edge and vehicles compared to that between cloud and vehicles. Considering the processing and latency tradeoffs between edge and cloud, an offloading algorithm is required. The underlying edge and cloud servers consume a high amount of energy while executing the computationally intensive and time-critical requests from vehicles. Consequently, it becomes crucial to address the issue of edge and cloud energy consumptions.

In our system model, each vehicle sends a request to the communicating RSU for execution. A request *i* $\left(1\leq i\leq m\right)$ is sent to the RSU as a request message, $rm_{i}$. The request message is represented as a tuple, $rm_{i}=\left\{L_{i},\;Size_{i},\;CPU_{i},\;T_{i}^{max},\;L_{i}^{max},Pr_{i}^{max},Speed_{j},\;\left(x_{i,j}^{source},y_{i,j}^{source}\right),\;\left(x_{i,j}^{destination},\;y_{i,j}^{destination}\right)\right\}$. $L_{i}$ is the length of the request *i* in terms of million instructions (MI), $Size_{i}$ is the size of request *i* in terms of bits, $CPU_{i}$ is the CPU utilization of *i*, $Speed_{i}$ is the speed of the vehicle, *j* $\left(1\leq j\leq n\right)$. $\left(x_{i,j}^{source},y_{i,j}^{source}\right)$ is the location (longitude, latitude), which we denote by the source of the vehicle *j* when submitting the request *i*, and $\left(x_{i,j}^{destination},\;y_{i,j}^{destination}\right)$ is the location of the vehicle’s destination. $T_{i}^{max}$ is the maximum tolerable deadline for the execution of *i*, $L_{i}^{max}$ is the maximum tolerable latency, and $Pr_{i}^{max}$ is the maximum tolerable processing time.

**Figure 2:**
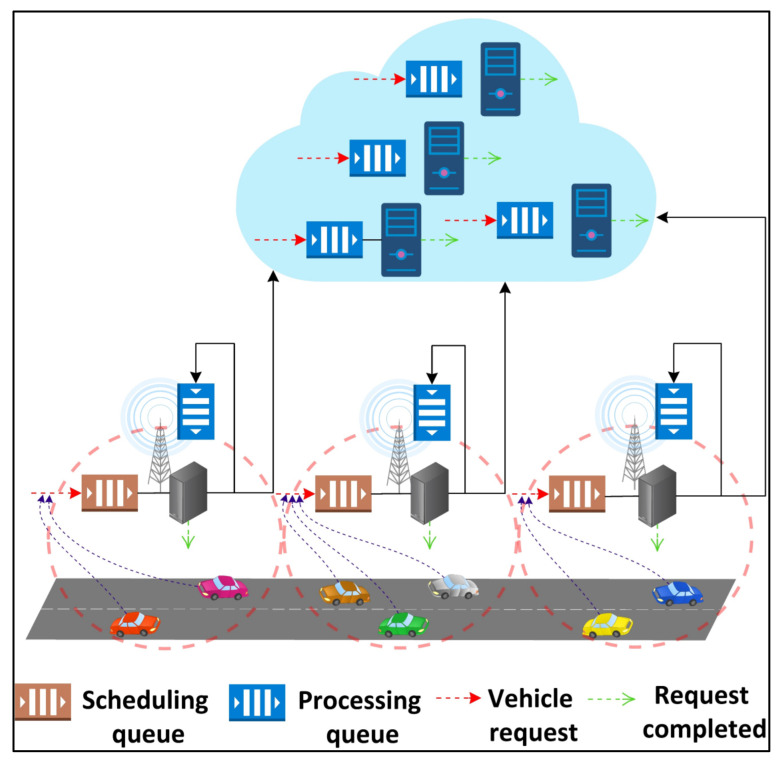
Request offloading and execution using scheduling and processing queues.

Each edge server consists of two queues, (1) scheduling and (2) processing, as shown in Figure 2. A vehicle’s request, when submitted, enters the scheduling queue of the connected RSU’s edge server. The scheduling queue decides to either execute the request locally on the server itself or offload it to one of the cloud servers. The offloading decision is made in such a way that the energy consumption of the request execution is minimized while respecting the SLA. If the decision is made to execute the request locally, then the request is submitted to the processing queue of the edge, else to the processing queue of the selected cloud server.

## 4. ESCOVE: Energy-SLA-Aware Edge–Cloud Computation Offloading in Vehicular Networks

In this section, we present our Energy-SLA-Aware Edge–cloud Computation Offloading in Vehicular Networks (ESCOVE) algorithm, which optimizes the energy consumption of vehicles’ requests while respecting requests’ SLA requirements. Consequently, ESCOVE decides whether to execute a request on an RSU’s edge server or one of the cloud servers.

ESCOVE consists of four steps: (1) calculating a request’s total execution time, latency, and processing time on edge and cloud servers, (2) calculating the corresponding energy consumptions on the servers, (3) scheduling requests to optimal servers in terms of energy while respecting the SLA, and (4) delivering request’s response to the submitter vehicle. This section describes the steps’ methods of computation.

### 4.1. Total Execution Time, Latency, and Processing Time Computation

In this step, ESCOVE computes a request’s total execution times, latencies, and processing times in case the request runs on the submitted edge server or one of the cloud servers. It then selects the server(s) where SLA is respected, i.e., the total execution time for request *i* is below $T_{i}^{max}$, latency is below $L_{i}^{max}$, and the processing time is below $Pr_{i}^{max}$.

#### 4.1.1. Total Execution Time, Latency, and Processing Time for Local (Edge) Computing

In this case, the scheduling queue of the edge server *k* $\left(1\leq k\leq o\right)$ decides to process a request *i* locally. Consequently, the total execution time $T_{i_{k}}^{total}$ for request *i* when executed locally on the edge server *k* is computed using Equation (1).

(1) $$T_{i_{k}}^{total}=T_{i_{j,k}}^{com}+T_{i_{k}}^{exec}+T_{r{\left(i\right)}_{k,l}}^{com}+T_{r{\left(i\right)}_{l,k+h}}^{com}+T_{r{\left(i\right)}_{k+h,j}}^{com}$$

where $T_{i_{j,k}}^{com}$ is the communication time required to transfer the request message $rm_{i}$ by the vehicle *j* to the edge server *k* (Equation (2)). $T_{r{\left(i\right)}_{k,l}}^{com}$ is the communication time needed to transfer the response of request *i* from edge server *k* to a cloud server *l* $\left(1\leq l\leq p\right)$ (Equation (3)). $T_{r{\left(i\right)}_{l,k+h}}^{com}$ is the communication time to transfer the response of the request *i* from *l* to the edge server *k + h* (Equation (3)). $T_{r{\left(i\right)}_{k+h,j}}^{com}$ is the communication time to transfer the response of the request *i* from *k* + *h* to *j* (Equation (4)). $T_{i_{k}}^{exec}$ is the time required to execute the request *i* by the edge server *k* (Equation (5)). After submitting the request *i* to the edge server *k*, the vehicle *j* might move outside *k*’s range depending on the destination and speed of *j*. Consequently, the location of *j*, while it will receive the response of *i*, is estimated by ESCOVE. The vehicle *j* will be under the range of RSU *k* + *h* while receiving the response, i.e., $h^{th}$ RSU after *k* on the source to destination path. The estimation of *h* is explained in Section 4.4

(2) $$T_{i_{j,k}}^{com}=\frac{Size_{rm_{i}}}{B_{j,k}}$$

(3) $$T_{r{\left(i\right)}_{k,l}}^{com}=T_{r{\left(i\right)}_{l,k+h}}^{com}=\frac{Size_{r\left(i\right)}}{B_{k,l}}$$

(4) $$T_{r{\left(i\right)}_{k+h,j}}^{com}=\frac{Size_{r\left(i\right)}}{B_{k+h,j}}$$

where $B_{j,k}$ is the communication bandwidth between vehicle *j* and RSU *k*,$\;B_{k,l}$ is the communication bandwidth between the RSU *k* and the cloud server *l*, and $B_{k+h,j}$ is the communication bandwidth between RSU *k* + *h* and vehicle *j*. A heterogeneous communication bandwidth between an RSU and the cloud servers is considered.

The time required by the edge server to execute the request is calculated using Equation (5).

(5) $$T_{i_{k}}^{exec}=T_{i_{k}}^{sched}+T_{i_{k}}^{proc}$$

where $T_{i_{k}}^{sched}$ is the time required to schedule the request *i* by the scheduling queue of edge server *k*. $T_{i_{k}}^{proc}$ is the time required to execute the request *i* by the processing queue of *k*. $T_{i_{k}}^{sched}$ is calculated using Equation (6),

(6) $$T_{i_{k}}^{sched}=T_{i_{k}}^{wait_{sched}}+T_{i_{k}}^{proc_{sched}}=T_{i_{k}}^{wait_{sched}}+c$$

where $T_{i_{k}}^{wait_{sched}}$ is the waiting time of *i* in the scheduling queue of the edge server *k* (Equation (7)) and $T_{i_{k}}^{proc_{sched}}$ is the processing time required to make the offloading decision by the scheduling queue. The value of $T_{i_{k}}^{proc_{sched}}$ is considered constant in the infrastructure under study. The waiting time in the scheduling queue is calculated as follows.

(7) $$T_{i_{k}}^{wait_{sched}}=\left\{\begin{array}{rr} 0, & \;\;i=1\\ \max\left(0,\;T_{{\left(i-1\right)}_{k}}^{sched}-IAT_{{\left(i,\;i-1\right)}_{k}}^{sched}\right), & \;\;i>1 \end{array}\right.$$

where $IAT_{{\left(i,\;i-1\right)}_{k}}^{sched}$ is the inter-arrival time between the requests *i* and *i* − 1 at the scheduling queue of the edge server *k*.

The processing time of request *i* at the processing queue of edge server *k* is calculated using Equation (8),

(8) $$T_{i_{k}}^{proc}=T_{i_{k}}^{wait_{proc}}+T_{i_{k}}^{proc_{proc}}$$

where $T_{i_{k}}^{wait_{proc}}$ is the waiting time of *i* in the processing queue of edge server *k* (Equation (9)) and $T_{i_{k}}^{proc_{proc}}\;$ is the request execution time in the processing queue of edge server *k* (Equation (10)). Consequently

(9) $$T_{i_{k}}^{wait_{proc}}=\left\{\begin{array}{rr} 0, & \;\;i=1\\ \max\left(0,\;T_{{\left(i-1\right)}_{k}}^{proc}-\left[T_{i_{k}}^{sched}-T_{i-1_{k}}^{sched}\right]\right), & \;\;i>1 \end{array}\right.$$

(10) $$T_{i_{k}}^{proc_{proc}}=\frac{L_{i}}{S_{k}}$$

where $S_{k}$ is the processing speed of the edge server *k* in million instructions per second (MIPS).

The latency of the request in the case of local computing is calculated using Equation (11) and the processing time is calculated using Equation (12).

(11) $$L_{i_{k}}=T_{i_{j,k}}^{com}+T_{r{\left(i\right)}_{k+h,j}}^{com}$$

(12) $$Pr_{i_{k}}=T_{i_{k}}^{proc}$$

#### 4.1.2. Total Execution Time, Latency, and Processing Time for Cloud Computing

In this case, the scheduling queue of edge server *k* decides to offload the request *i* to the cloud and process it on the cloud server *l*. The total execution time $T_{i_{l}}^{total}$ for request *i* when offloaded and executed on *l* is computed using Equation (13).

(13) $$T_{i_{l}}^{total}=T_{i_{j,k}}^{com}+T_{i_{k,l}}^{exec}+T_{r{\left(i\right)}_{l,k+h}}^{com}+T_{r{\left(i\right)}_{k+h,j}}^{com}$$

where $T_{i_{k,l}}^{exec}$ is the time required to execute the request *i* on *l* when offloaded by the edge server *k* (Equation (14)).

(14) $$T_{i_{k,l}}^{exec}=T_{i_{k}}^{sched}+T_{i_{l}}^{proc}$$

where $T_{i_{l}}^{proc}$ is the time required to execute *i* by the processing queue of *l*. It is calculated using Equation (15).

(15) $$T_{i_{l}}^{proc}=T_{i_{l}}^{wait_{proc}}+T_{i_{l}}^{proc_{proc}}$$

where $T_{i_{l}}^{wait_{proc}}$ is the waiting time of *i* in the processing queue of *l* (Equation (16)) and $T_{i_{l}}^{proc_{proc}}$ is the request execution time in the processing queue of *l* (Equation (17)).

(16) $$T_{i_{l}}^{wait_{proc}}=\left\{\begin{array}{rr} T_{i_{k,l}}^{com}, & \;\;i=1\\ \max\left(0,\;T_{{\left(i-1\right)}_{l}}^{proc}-\left[T_{i_{k}}^{sched}-T_{i-1_{k}}^{sched}\right]\right)+T_{i_{k,l}}^{com}, & \;\;i>1 \end{array}\right.$$

(17) $$T_{i_{l}}^{proc_{proc}}=\frac{L_{i}}{S_{l}}$$

where $T_{i_{k,l}}^{com}$ is the communication time required to transfer the request message of *i* from edge server *k* to *l* (Equation (18)) and $S_{l}$ is the processing speed of the cloud server *l* in MIPS.

(18) $$T_{i_{k,l}}^{com}=\frac{Size_{rm_{i}}}{B_{k,l}}$$

The latency of the request when offloaded to the cloud is calculated using Equation (19) and the processing time is calculated using Equation (20).

(19) $$L_{i_{l}}=T_{i_{j,k}}^{com}+T_{r{\left(i\right)}_{k+h,j}}^{com}$$

(20) $$Pr_{i_{l}}=T_{i_{l}}^{proc}$$

### 4.2. Energy Consumption Computation

In this step, ESCOVE computes the energy consumption of a request’s execution on the servers where the SLA is respected. The energy consumptions for making the offloading decision and for the communication of request and response are not considered in this paper. The energy consumed for processing request *i* is given by Equation (21).

(21) $$E_{i_{x}}^{proc}=P_{i_{x}}^{proc}\times T_{i_{x}}^{proc_{proc}}\;x\in \left\{k\;\cup \;\left\{1,\;2,\;3,\;\ldots ,\;p\right\}\right\}\;s.t.\;T_{i_{x}}^{total}\leq T_{i}^{max},L_{i_{x}}\leq L_{i}^{max},\;and\;Pr_{i_{x}}\leq Pr_{i}^{max}\;$$

where $P_{i_{x}}^{proc}$ is the power consumed by server *x* while processing the request *i*. We use locally corrected linear regression (LC-LR) [14] to estimate the power consumption of server *x* while executing request *i*. LC-LR is the summation of the classical linear regression model and an error correction term as stated in Equation (22).

(22) $$P_{i_{x}}^{proc}=P_{CPU_{i_{x}}}^{\prime }+{Ø}_{CPU_{i_{x}}}$$

where $P_{CPU_{i_{x}}}^{\prime }$ is the predicted power consumption for request *i* using the linear regression power model (Equation (23)) for the server *x* and ${Ø}_{CPU_{i_{x}}}$ is the error correction term for *i* on server *x* (Equation (24)). In this paper, we consider a power prediction model based only on the CPU utilization of the server. This is because the CPU is considered to be the most dominant power consumer in a computing server [5].

(23) $$P_{CPU_{i_{x}}}^{\prime }=\alpha_{x}+\left(\beta_{x}\times CPU_{i}\right)$$

where $\alpha_{x}\;$ and $\beta_{x}$ are the regression coefficients of the linear regression model for server *x*. The coefficients’ values for a server *x* are obtained using a training dataset consisting of the CPU utilization values and the corresponding power consumption values. The error correction term corresponding to $CPU_{i}$ is calculated, as stated in Equation (24), by constructing a linear line between the CPU utilization values $CPU^{\prime }$ and $CPU^{\prime \prime }\;$ from the training dataset such that $CPU^{\prime }\leq CPU_{i}\leq CPU^{\prime \prime }$.

(24) $${Ø}_{CPU_{i_{x}}}=e_{CPU^{\prime }}+\frac{\left(e_{CPU^{\prime \prime }}-e_{CPU^{\prime }}\right)\left(CPU_{i}-CPU^{\prime }\right)}{\left(CPU^{\prime \prime }-CPU^{\prime }\right)}$$

where $e_{CPU^{\prime }}$ is the intercept and $\frac{\left(e_{CPU^{\prime \prime }}-e_{CPU^{\prime }}\right)}{\left(CPU^{\prime \prime }-CPU^{\prime }\right)}$ is the slope of the constructed linear model.

### 4.3. Request Scheduling and Execution

In this step, ESCOVE will schedule a request to the server having the minimum request execution energy consumption (Equation (21)) among those where the request’s SLA is respected. The request will be executed on the scheduled server. If no server can satisfy the request’s SLA requirements, then the request is processed on the server where the total execution time is minimized.

### 4.4. Request-Response Delivery

**Figure 3:**
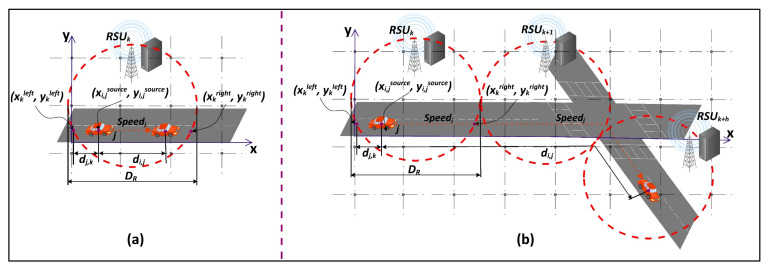
(**a**) Request-response delivery when the vehicle is in the range of the RSU to which the request was submitted. (**b**) Request-response delivery when the vehicle is not in the range of the RSU to which the request was submitted.

In this step, ESCOVE will send a request-response back to the vehicle after a request has been executed. The algorithm first estimates the position of the vehicle *j* while receiving the request-response. The vehicle can be in the range of RSU *k* where the request was submitted (Figure 3a) or it can be out of the range of *k* (Figure 3b). To determine the position of *j*, the server *x* executing the request will calculate: (1) the distance $d_{j,k}$ that *j* traveled in the range of *k* before submitting the request *i* (Equation (25)), and (2) distance $d_{i,j}$ traveled by *j* during the time the request is being executed (Equation (26)).

(25) $$d_{j,k}=\left\{\begin{array}{c} x_{i,j}^{source}-x_{k}^{left},\;\;x_{i,j}^{source}<x_{i,j}^{destinaion}\\ x_{k}^{right}-x_{i,j}^{source},\;\;x_{i,j}^{source}>x_{i,j}^{destinaion} \end{array}\right.$$

(26) $$d_{i,j}=Speed_{j}\times T_{i_{k}}^{total}$$

The vehicle *j* will be in the range of server *k* if $d_{j,k}+d_{i,j}\leq D_{R}$, otherwise, it will be outside the range of *k*. If *j* is in the range of *k* and *i* is executed by *k*, then the request-response will be directly transmitted from *k* to *j*. If *j* is in the range of *k* and *i* is executed by *l*, then the request-response will be transmitted from *l* to *k* and then from *k* to *j*. If *j* is outside the range of *k*, then the algorithm will determine the value of *h*, such that the vehicle will be in the range of RSU *k* + *h* while receiving the response. The value of *h* is computed using Equation (27).

(27) $$h=⎡\frac{d_{i,j}-\left(D_{R}-d_{j,k}\right)}{D_{R}}⎤$$

The objective of the ESCOVE algorithm is thus defined as the minimization of the energy consumption (Equation (28)) subject to the following constraints: (1) the total execution time of the request should be less than or equal to the maximum permissible delay (deadline) (Equation (29)), (2) the request’s latency should be less than or equal to the maximum permissible latency (Equation (30)), and (3) the request’s processing time should be less than or equal to the maximum permissible processing time (Equation (31)).

***Objective***:

(28) $$Minimize\;\left(E_{i_{x}}^{proc}\right),\;x\;\in \;\left\{k\;\cup \;\left\{1,\;2,\;3,\;\ldots ,\;p\right\}\right\}$$

***Constraints***:

(29) $$T_{i_{x}}^{total}\leq T_{i}^{max}\;$$

(30) $$L_{i_{x}}\leq L_{i}^{max}$$

(31) $$Pr_{i_{x}}\leq Pr_{i}^{max}$$

where $T_{i}^{max}$ is the maximum delay for request i to be serviced, $L_{i}^{max}$ and $Pr_{i}^{max}$ are the maximum latency and processing required for QoS respectively.

ESCOVE algorithm offloads vehicle’s requests as follows:

⮚ A vehicle submits a request to the RSU which is in the vehicle’s range.
⮚ The RSU’s edge server computes the latency, processing and total execution times of the request on itself and all the cloud servers.
⮚ The edge server selects a group of the servers where the request’s constraints, i.e., latency, processing time, and deadline, are satisfied. If no server satisfies the constraints, then the request will be offloaded for execution to the server resulting in the minimum total execution time.
⮚ If there exists a group of servers satisfying the constraints, then the edge server computes the energy consumption for processing the request on each of the selected servers in the group.
⮚ The edge server then offloads the request to the server that has the minimum energy consumption.
⮚ The server which is processing the request estimates the location of the vehicle at the time the vehicle receives the response. It then sends the response to the vehicle’s corresponding RSU.

**Figure 4:**
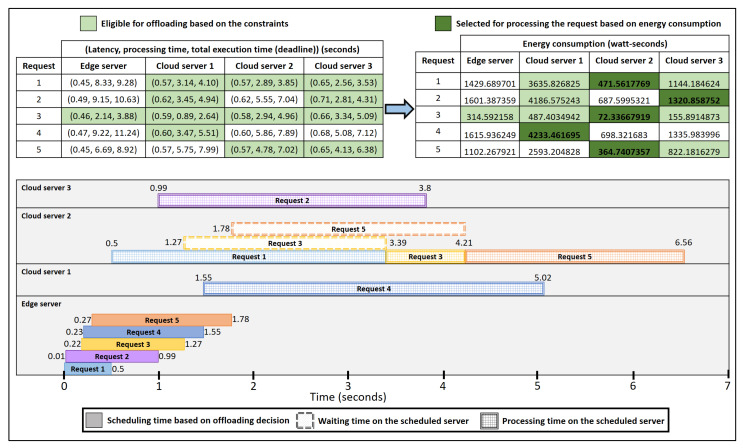
Gantt chart representing the offloading of requests using ESCOVE.

Figure 4 shows the Gantt chart of offloading requests using ESCOVE. In this example, the vehicular network consists of one edge and three cloud servers. The specifications of requests are stated in Table 1. In the example, the latency, processing time, and deadline requirements for requests are considered as 1 s, 5 s, and 10 s respectively. The power consumption values of requests on the edge and cloud servers are presented in Table 2.

When a vehicle submits request 1 to the edge server, a request enters the scheduling queue of the server. The server computes the latency, processing and total execution times of the request on itself and all the cloud servers. A group of servers is then selected where the SLA requirements are satisfied, i.e., cloud server 1, cloud server 2, and cloud server 3, as represented in Figure 4. The edge server then computes the energy consumption of processing the request on the selected group. The request is then offloaded to the server where the energy consumption is minimized, i.e., the cloud server 2 (Figure 4). The time to make the offloading in the scheduling queue of the edge server is considered 0.5 s. Requests 2, 3, 4, and 5 are offloaded similarly.

As shown in the figure, request 1 is offloaded to the cloud server 2 at 0.5 s after the offloading decision is made by the edge server. The processing of request 1 on cloud server 2 ends at 3.39 s. When request 2 arrives at the scheduling queue of the edge server at 0.01 s, it waits in the queue as request 1 is being processed. The offloading decision for request 2 is made at 0.99 s by the edge server, and the request is offloaded to cloud server 3. The processing of request 2 ends at 3.8 s. Similarly, request 3 is offloaded at 1.27 s by the edge server to cloud server 2. As request 1 is still being processed at cloud server 2, request 3 will wait in the processing queue of the server. Once the processing of request 1 completes, the one for request 3 starts at 3.39 s and lasts till 4.21 s. Request 4 is offloaded to cloud server 1 by the edge server at 1.55 s. Request 5 is offloaded to cloud server 2 at 1.78 s. Request 5 waits in the processing queue of the cloud server 3 till requests 1 and 2 are processed.

**Figure 5:**
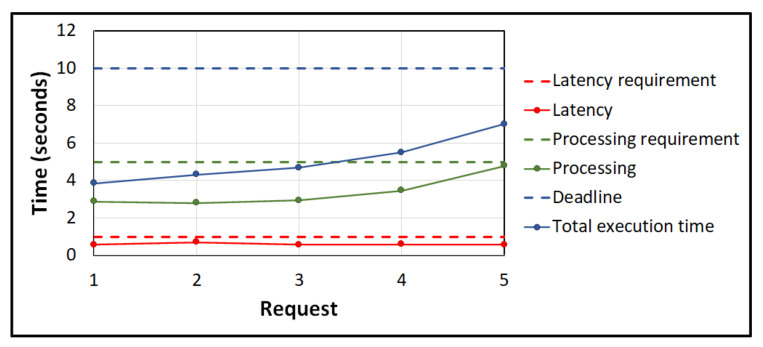
Latency, processing and total execution times of requests using ESCOVE.

Figure 5 shows the latency, processing and total execution times for the five requests used in the example. In addition, the latency, processing time, and deadline requirements are also represented. The figure shows that all the three SLA requirements are satisfied using the ESCOVE algorithm.

## 5. Performance Evaluation

In this section, we describe the experimental environment and the experiments performed to evaluate our proposed algorithm, ESCOVE. We then analyze and give insights on the obtained numerical results. We evaluate the performance of ESCOVE in a heterogeneous edge–cloud vehicular network in terms of energy consumption, latency, processing time, execution time, and percentage of SLA violations (SLAVs).

### 5.1. Experimental Environment

To evaluate the performance of ESCOVE, we create a heterogeneous edge–cloud vehicular network consisting of 10 RSUs (edge servers) of 3 different types and 20 cloud servers of 3 different types. The network is implemented using MATLAB 2020a [15]. The specifications of the servers’ types are presented in Table 3. Servers 1 and 2 from the edge servers are part of our Intelligent Distributed Computing and Systems (INDUCE) research laboratory at the College of Information Technology of the United Arab Emirates University. The specifications of servers 3–6 are taken from the SPEC Power benchmark suite [16] in a way that they belong to the same family of the servers present in the laboratory, but with different architectures and resource capabilities.

To develop the LC-LR power model for each server type, we use a dataset consisting of CPU utilization values of a server and the corresponding power consumption. For servers 1 and 2, we run CPU Load Generator [21] to stress the CPU and measure the values of CPU utilization and corresponding power consumption. CPU Load Generator is a tool that allows generating a fixed CPU load for a finite user-defined duration. To measure the values of power consumption, we use a 4-channel digital oscilloscope, Tektronix—TBS2000 100 MHz with 1 GS/s of sampling [22]. We connect the oscilloscope to a current probe [23] and a voltage probe [23] to measure the server’s current and voltage respectively. The current and voltage values are extracted from the oscilloscope using the LabVIEW program [24]. The power consumption is then computed as the product of current and voltage. For servers 3–6, the values of CPU utilization and corresponding power consumption are obtained from SPEC Power.

To obtain the position of vehicles in the simulated network, we use the Vehicle-Crowd Interaction (VCI)—DUT dataset [25]. We use the x_est and y_est columns of the dataset, representing the estimated vehicles’ position, as the source location of the vehicles in our experiments. Table 4 shows the experimental parameters. The value for RSU transmission power for the MEES algorithm is based on the literature [10]. The latency requirement [26] and the processing time and deadline requirements [27] for the request are based on the literature. 

### 5.2. Experiments

The set of experiments performed to obtain the CPU utilization and power consumption datasets for servers 1 and 2, and to simulate the edge–cloud vehicular network for implementing ESCOVE are explained in this section.

To obtain the datasets for serves 1 and 2, we stress the CPU load generator on each server to generate a CPU load between 0–100% for five minutes at an interval of 10%. We measure the CPU utilization and the corresponding power consumption values for each generated CPU load at every second and write them to a file. The values are then averaged. We repeat the experiment for each CPU load five times and average all the averages. Table 5 shows the values of CPU utilization and corresponding power consumption for servers 1–6.

**Figure 6:**
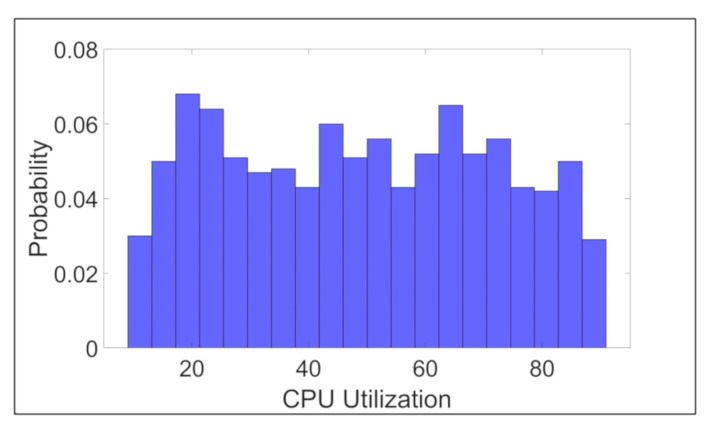
Probability distribution of the generated requests’ CPU-utilization values.

**Figure 7:**
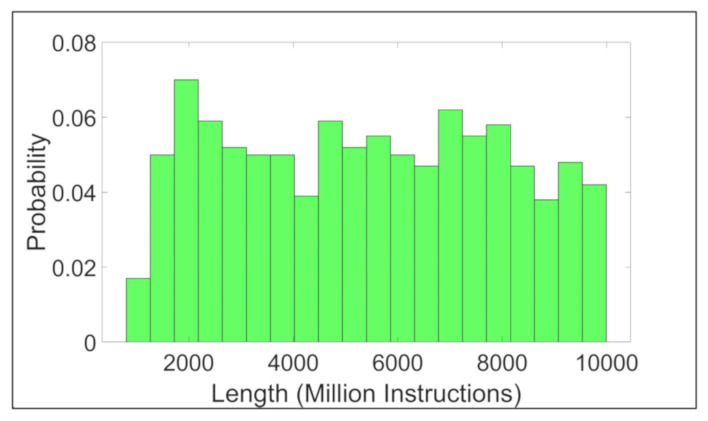
Probability distribution of the generated requests’ length values.

To evaluate the performance of ESCOVE, we simulate an edge–cloud vehicular network with 1000 vehicles [28]. The network consists of 10 edge servers, with edge servers’ types (Table 1) equally distributed, and 20 cloud servers, with cloud servers’ types (Table 1) equally distributed. For the source locations of the vehicles, we take the first 1000 locations from the VCI—DUT dataset. For the geographical location of the RSUs (edge servers), we randomly generate the x and y coordinates between the minimum and the maximum x and y coordinates values of the vehicles in a way that the RSUs are equidistant from each other. Each vehicle in the network generates a request. Requests arrive at the edge servers with a mean arrival rate of 10 requests per second [29]. For each vehicle’s request, we randomly generate a CPU-utilization value and request length (MI). CPU utilization is generated between the minimum and maximum CPU-utilization values. The length is generated between the minimum and maximum request-length values. Figure 6 shows the probability distribution of the CPU utilization values for the generated requests. Figure 7 shows the probability distribution of the generated requests’ lengths.

In our experiments, for each vehicle’s request arriving at the connected edge server, the server computes the energy consumption of requests on all servers (edge and the cloud) where requests’ SLA requirements are satisfied. A request is then scheduled for execution on the server where the energy consumption is minimized. If none of the servers can satisfy the SLA requirements, then the request will be scheduled to the server where the total execution time is minimized. We measure the energy consumption, latency, processing time, and total execution time for each request. We also compute the total energy consumption as the sum of the energy consumption of each request and the percentage of SLAVs (Equation (32)).

(32) $$\%\;SLAVs=\frac{\overset{m}{\underset{i=1}{\#}}\left(\left[T_{i_{x}}^{total}>T_{i}^{max}\right]\;\left|\left|\;\left[L_{i_{x}}>L_{i}^{max}\right]\;\right|\right|\;\left[Pr_{i_{x}}>Pr_{i}^{max}\right]\right)}{m}$$

To demonstrate the performance of ESCOVE, we compare it with a state-of-the-art algorithm:

- **MEC-enabled Energy-Efficient Scheduling (MEES)**: A scheduling scheme that schedules the vehicles’ requests either to the communicating edge server or offloads them to other edge servers via multi-hop requests transmission. The scheduling decision is made in a way that the energy consumption of the edge servers is minimized under deadline constraints. If no edge servers satisfy the SLA requirements for a request, then the request is scheduled to the server providing the minimum execution time.

In addition, we compare the performance of ESCOVE with the following two approaches:

1. **Random Non-SLA-Aware (RNSA) offloading**: An offloading scheme that schedules the vehicles’ requests randomly to the edge server (to which a vehicle submits the request) or a cloud server without considering the SLA constraints.
2. **Random SLA-Aware (RSA) offloading**: An offloading scheme that schedules the vehicles’ requests randomly to the edge server (to which a vehicle submits the request) or a cloud server considering the SLA constraints.

We repeat the experiments with MEES, RSA, and RNSA approaches, and measure the energy consumption of each request, latency, processing and total execution times for each request, total energy consumption, and percentage of SLAVs.

### 5.3. Experimental Results Analysis

In this section, we analyze and compare the performance of our proposed ESCOVE offloading algorithm with the MEES, RSA, and RNSA approaches.

**Figure 8:**
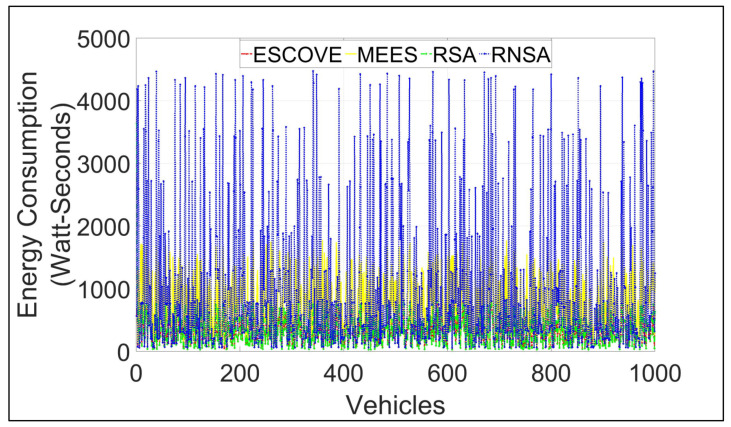
Energy consumption of requests using ESCOVE, MEES, RSA, and RNSA.

Figure 8 shows the energy consumption of the vehicles’ requests using ESCOVE, MEES, RSA, and RNSA. It shows that the overall energy consumption of ESCOVE is minimized. This is because of the energy optimization objective of ESCOVE that schedules the vehicles’ requests in a way that the energy consumption for each request’s execution is minimized. The energy consumption of MEES is more than that of ESCOVE as MEES schedules requests only on the edge servers without involving the cloud. Consequently, the processing of requests requires more time using MEES, leading to higher energy consumption. In addition, the energy consumption of RSA is less than that of MEES, even though the former is non-energy-efficient. This is because of the inclusion of the cloud in RSA for processing requests, leads to less time and thus less energy consumption. The energy consumption of RNSA is the maximum. This is because RNSA randomly schedules requests without considering the SLA. Consequently, a request’s execution time in RNSA is greater compared with MEES and RSA. This leads to higher energy consumption, as the energy consumed by a request is a function of its execution time.

**Figure 9:**
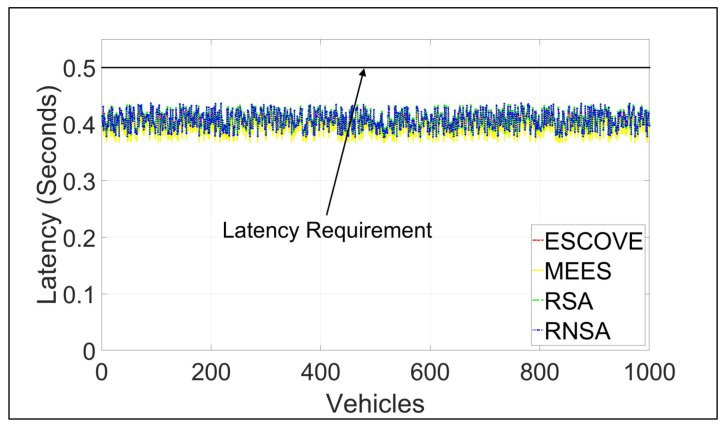
Latencies of requests using ESCOVE, MEES, RSA, and RNSA.

Figure 9 shows the latencies of requests using ESCOVE, MEES, RSA, and RNSA. It shows that latencies using all the algorithms are below the permissible latency requirement of 500 milliseconds. The figure shows that requests’ latencies using the MEES are lower compared to other algorithms. This is because, MEES considers only the edge layer for scheduling requests, while ESCOVE, RSA, and RNSA consider both edge and cloud layers. The inclusion of a cloud layer in the latter results in higher latency.

**Figure 10:**
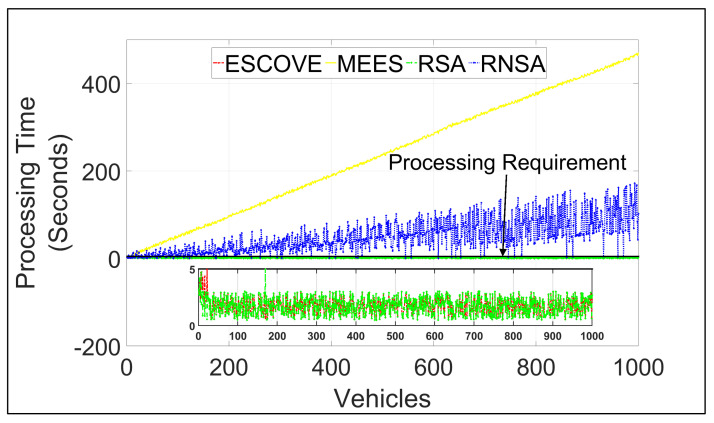
Processing times of requests using ESCOVE, MEES, RSA, and RNSA.

Figure 10 shows requests’ processing times using ESCOVE, MEES, RSA, and RNSA. It shows that processing times for all requests using ESCOVE and RSA are below the maximum permissible processing time of 5 s. This is because of the processing time constraint consideration in the algorithms while scheduling requests. A zoomed-in image for the processing times using ESCOVE and RSA has been presented in the figure for clarity. Regarding the MEES and RNSA, not all requests meet the processing time constraint. This is because, the requirement is not considered in RNSA, while for the MEES only the edge servers are not able to respect the processing time requirement. The processing time in MEES increases with increasing vehicles as requests’ waiting times increases.

**Figure 11:**
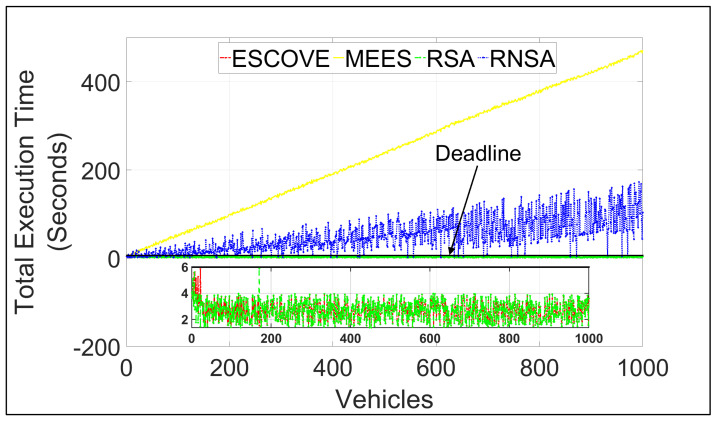
Total execution times of requests using ESCOVE, MEES, RSA, and RNSA.

Figure 11 shows the total execution time of requests using ESCOVE, MEES, RSA, and RNSA. It shows that execution times for all requests using ESCOVE and RSA are below the acceptable deadline. This is because of the deadline constraint considered in the algorithms while scheduling requests. The execution times of ESCOVE and RSA are zoomed in for clarity in the figure. MEES and RSA do not satisfy the deadline constraint. This is because for MEES, the edge servers are not able to process requests in the given deadline, and for RSA the deadline constraint is not considered. The execution using MEES increases with increasing vehicles due to increasing waiting time at the edge servers.

**Figure 12:**
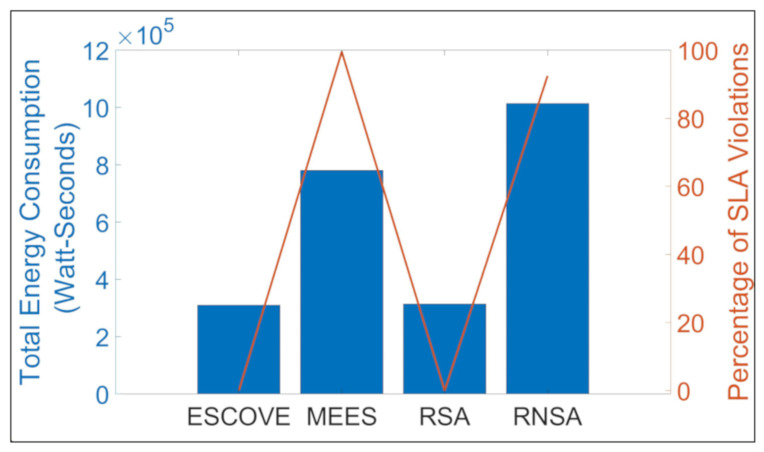
Total energy consumption and percentage SLAVs using ESCOVE, RNSA, and RSA.

**Figure 13:**
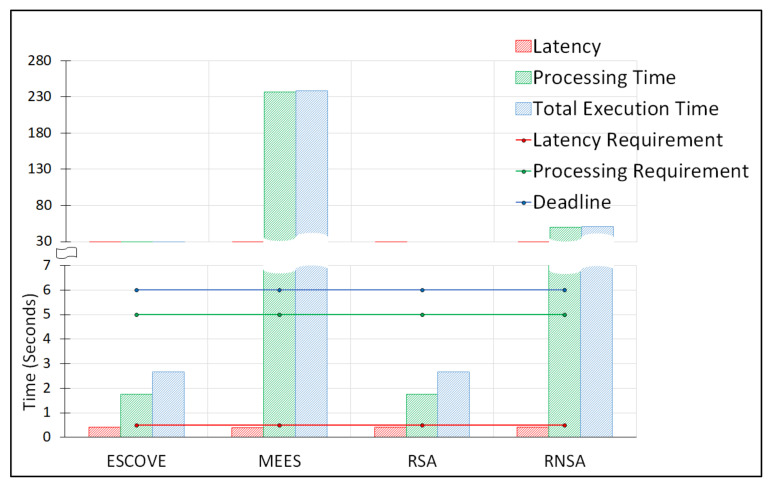
Average latency, processing and total execution times using ESCOVE, MEES, RSA, and RNSA.

Figure 12 shows the total energy consumption of all requests and percentage of SLAVs using ESCOVE, MEES, RSA, and RNSA. It shows that ESCOVE has the least energy consumption with 0% of SLAVs. The SLAVs using RSA is also 0%, but the energy consumption is more compared to that of ESCOVE. The energy consumption of MEES is higher than that of ESCOVE and RSA, and the percentage SLAVs is 99.5%. The RNSA approach has the worst performance in terms of total energy consumption. The percentage SLAVs using RNSA is 92.4%. Figure 13 shows the average latency, processing and total execution times for the algorithms. It shows that the average latency of all the algorithms is below the permissible latency requirement. Regarding the processing and total execution times, the values for ESCOVE and RSA are below the corresponding permissible limit. However, for MEES and RNSA, the processing time and deadline constraints are not satisfied.

In summary, ESCOVE saves 60.26% of total energy consumption compared to MEES, 1.47% compared to RSA, and 69.44% compared to RNSA, with no SLAVs.

## 6. Conclusions

Energy-efficient computation offloading is important in edge–cloud vehicular networks for executing computationally intensive and time-critical vehicles’ requests respecting the SLA. In this paper, we propose the Energy-SLA-Aware Edge–Cloud Computation Offloading in Vehicular Networks (ESCOVE) algorithm. The proposed algorithm schedules a vehicle’s request to either an edge server or to a cloud server in a way that the energy consumed to execute the request is minimized and the request’s SLA requirements are satisfied. To the best of our knowledge, we are the first ones to propose an energy-SLA-aware offloading algorithm that optimizes the energy consumption of the edge as well as the cloud servers, considering latency, processing time, and deadline constraints. We compared the performance of ESCOVE with one state-of-the-art algorithm, MEES, and two offloading approaches, RNSA and RSA, in terms of a request’s energy consumption, latency, processing and total execution times, total energy consumption for all requests, and the percentage of SLAVs. Our experimental results reveal that ESCOVE outperforms the state-of-the-art algorithm and other approaches in terms of energy consumption as well as SLAVs.

## Figures and Tables

**Table 1:** Specifications of requests used in the example.

Request	Arrival Time (s)	CPU Utilization (%)	Length (MI)	Size (Bits)
1	0	75.17%	8332.5	2.59
2	0.009894565	82.46%	9152.1	12.33
3	0.216261766	20.15%	2142.9	4.97
4	0.225322547	83.07%	9220.4	8.45
5	0.271152309	60.58%	6691.2	2.65

**Table 2:** Power consumption values of requests on the edge and cloud servers used in the example.

Request	Power Consumption (Watts)			
	Edge Server	Cloud Server 1	Cloud Server 2	Cloud Server 3
1	171.5796508	1199.940949	169.7789491	480.6048427
2	174.9743293	1257.967833	225.3900649	505.1290714
3	146.8080155	625.4940858	101.270207	254.6198867
4	175.2569607	1262.639527	227.2102055	507.131226
5	164.7331494	1065.769649	163.5307232	430.0605889

**Table 3:** Specifications of the servers’ types used in the experiments.

Location	Server	Description	MIPS
edge	1	Sun Fire Intel_Xeon CPU core of 2.80 GHz, Dual-core, with 512 KB of cache and 4 GB of memory for each core, CPU voltage rating 1.5 V, OS version CentOS 6.8(i686)	1000
	2	Sun Fire X4100 with AMD_Operaton252 CPU of 2.59 GHz, dual CPU, single-core, with 1 MB of cache and 2 GB of memory for each core, CPU voltage rating of 3.3–2.9 V, OS version Red Hat Enterprise Linux Server release 7.3 (Mapio)	1200
	3	Dell Inc. PowerEdge R260 with Intel Xeon E5-2670 CPU core of 2.6 GHz CPU, 8 cores, with 2 MB cache, 4 GB 2Rx8 PC3L10600E-9 ECC memory, and 1 × 100 GB SATA SSD disk drive [17]	1450
cloud	4	SGI Rackable C2112-4G10 with AMD Opteron 6276 CPU core of 2.30 GHz, 16 cores, 4 GB 2Rx8 PCL-10600R memory and 1 × 120 GB 2.5” SSD SATA disk drive [18]	2750
	5	Hewlett Packard Enterprise ProLiant DL360 Gen9 with Intel Xeon E5-2699 v3 CPU core of 2.30 GHz, 18 cores, with 45 MB L3 Cache, 8 GB 2Rx8 PC4-2133P memory, and 1 × 400 GB SSD SATA disk drive [19]	3000
	6	Acer Incorporated Acer AR585 F1 with AMD Opteron 6238 CPU core of 2.60 GHz, 12-core, with 16 MB L3 cache, 4 GB 2Rx8 PC3L-10600E memory, and 1 × 500 GB SATA2 7200 RPM 3.5” HDD disk drive [20]	3500

**Table 4:** Experimental parameters.

Parameter	Value
number of vehicles (*n*)	1000
number of edge servers (*o*)	10
number of cloud servers (*p*)	20
request’s minimum CPU utilization	10%
request’s maximum CPU utilization	90%
minimum request length	1000 MI
maximum request length	10,000 MI
request latency	500 milliseconds
request processing time	5 s
request deadline	6 s
vehicle—RSU bandwidth	350 megabits/second
RSU—cloud bandwidth	uniform (500, 1000) megabits/second
request’s men arrival rate	10 requests/second
RSU transmission power	uniform(0.01, 1) watts

**Table 5:** CPU utilization and corresponding power consumption for the servers used in the experiments.

CPU Utilization (%)	Power Consumption (Watts)					
	Server 1	Server 2	Server 3	Server 4	Server 5	Server 6
0	138.2685	204.2420	54.1	265	45	127
10	142.2829	204.9672	78.4	531	83.7	220
20	146.7379	205.9185	88.5	624	101	254
30	151.1429	206.6314	99.5	718	118	293
40	155.3824	207.5923	115	825	133	339
50	159.9734	208.5179	126	943	145	386
60	164.4558	209.1885	143	1060	162	428
70	169.1667	210.2377	165	1158	188	463
80	173.8268	211.1731	196	1239	218	497
90	178.4852	211.8091	226	1316	248	530
100	181.7913	214.9755	243	1387	276	559

## References

[1] Hartenstein H., Laberteaux L.P. (2008). A tutorial survey on vehicular ad hoc networks. IEEE Commun. Mag..

[2] Mell P., Grance T. (2011). The NIST Definition of Cloud Computing Recommendations of the National Institute of Standards and Technology. Nist Spec. Publ..

[3] Xie R., Tang Q., Wang Q., Liu X., Yu F.R., Huang T. (2019). Collaborative Vehicular Edge Computing Networks: Architecture Design and Research Challenges. IEEE Access.

[4] Ismail L., Materwala H. IoT-Edge-Cloud Computing Framework for QoS-Aware Computation Offloading in Autonomous Mobile Agents: Modeling and Simulation. Proceedings of the International Conference on Mobile, Secure, and Programmable Networking.

[5] Ismail L., Materwala H. (2018). Energy-Aware VM Placement and Task Scheduling in Cloud-IoT Computing: Classification and Performance Evaluation. IEEE Internet Things J..

[6] Zhang K., Mao Y., Leng S., Maharjan S., Zhang Y. Optimal Delay Constrained Offloading for Vehicular Edge Computing Networks. Proceedings of the IEEE International Conference on Communications (ICC).

[7] Du J., Yu F.R., Chu X., Feng J., Lu G. (2019). Computation Offloading and Resource Allocation in Vehicular Networks Based on Dual-Side Cost Minimization. IEEE Trans. Veh. Technol..

[8] Zhao J., Li Q., Gong Y., Zhang K. (2019). Computation offloading and resource allocation for cloud assisted mobile edge computing in vehicular networks. IEEE Trans. Veh. Technol..

[9] Sun F., Hou F., Cheng N., Wang M., Zhou H., Gui L., Shen X. (2018). Cooperative Task Scheduling for Computation Offloading in Vehicular Cloud. IEEE Trans. Veh. Technol..

[10] Ning Z., Huang J., Wang X., Rodrigues J.J.P.C., Guo L. (2019). Mobile Edge Computing-Enabled Internet of Vehicles: Toward Energy-Efficient Scheduling. IEEE Netw..

[11] Huang X., He L., Zhang W. Vehicle Speed Aware Computing Task Offloading and Resource Allocation Based on Multi-Agent Reinforcement Learning in a Vehicular Edge Computing Network. Proceedings of the 2020 IEEE International Conference on Edge Computing (EDGE).

[12] Huang X., Xu K., Lai C., Chen Q., Zhang J. (2020). Energy-efficient offloading decision-making for mobile edge computing in vehicular networks. EURASIP J. Wirel. Commun. Netw..

[13] Pu L., Chen X., Mao G., Xie Q., Xu J. (2019). Chimera: An Energy-Efficient and Deadline-Aware Hybrid Edge Computing Framework for Vehicular Crowdsensing Applications. IEEE Internet Things J..

[14] Ismail L., Abed E.H. (2019). Linear Power Modeling for Cloud Data Centers: Taxonomy, Locally Corrected Linear Regression, Simulation Framework and Evaluation. IEEE Access.

[15] MATLAB Documentation. https://www.mathworks.com/help/matlab/.

[16] SPECpower_ssj2008. https://www.spec.org/power_ssj2008/results/res2007q4/power_ssj2008-20071128-00004.html.

[17] Server 3: SPECpower_ssj2008. https://www.spec.org/power_ssj2008/results/res2012q1/power_ssj2008-20120306-00434.html.

[18] Server 4: SPECpower_ssj2008. https://www.spec.org/power_ssj2008/results/res2012q1/power_ssj2008-20120306-00437.html.

[19] Server 5: SPECpower_ssj2008. https://www.spec.org/power_ssj2008/results/res2016q1/power_ssj2008-20151215-00708.html.

[20] Server 6: SPECpower_ssj2008. https://www.spec.org/power_ssj2008/results/res2012q1/power_ssj2008-20120213-00420.html.

[21] Carlucci G. CPU Load Generator. https://github.com/GaetanoCarlucci/CPULoadGenerator.

[22] Tektronix TBS 2000 Digital Oscilloscope. https://download.tek.com/manual/TBS2000-User-RevC-EN-077114701.pdf.

[23] Wedlock B.D., Roberge J. (1969). Electronic Components and Measurements.

[24] What is LabVIEW?—National Instruments. https://www.ni.com/en-lb/shop/labview.html.

[25] Yang D., Li L., Redmill K., Özgüner Ü. Top-view trajectories: A pedestrian dataset of vehicle-crowd interaction from controlled experiments and crowded campus. Proceedings of the 2019 IEEE Intelligent Vehicles Symposium (IV).

[26] Jian Z., Muqing W., Min Z. (2019). Joint computation offloading and resource allocation in C-RAN with MEC based on spectrum efficiency. IEEE Access.

[27] He Y., Zhai D., Huang F., Wang D., Tang X., Zhang R. (2021). Joint Task Offloading, Resource Allocation, and Security Assurance for Mobile Edge Computing-Enabled UAV-Assisted VANETs. Remote Sens..

[28] Di Maio A., Soua R., Palattella M.R., Engel T. ROADNET: Fairness-and throughput-enhanced scheduling for content dissemination in VANETs. Proceedings of the 2018 IEEE International Conference on Communications Workshops (ICC Workshops).

[29] Ma X., Zhang J., Yin X., Trivedi K.S. (2011). Design and analysis of a robust broadcast scheme for VANET safety-related services. IEEE Trans. Veh. Technol..

